# The Mesencephalic Trigeminal Nucleus Controls Food Intake and Body Weight via Hindbrain POMC Projections

**DOI:** 10.3390/nu13051642

**Published:** 2021-05-13

**Authors:** Samantha M. Fortin, Jack Chen, Harvey J. Grill, Matthew R. Hayes

**Affiliations:** 1 Department of Psychiatry, Perelman School of Medicine, University of Pennsylvania, Philadelphia, PA 19104, USA; safortin@pennmedicine.upenn.edu (S.M.F.); jack.chen@pennmedicine.upenn.edu (J.C.); 2 Department of Psychology, University of Pennsylvania, Philadelphia, PA 19104, USA; grill@psych.upenn.edu

**Keywords:** obesity, melanocortin, MC4R

## Abstract

The mesencephalic trigeminal nucleus (Mes5) processes oral sensory–motor information, but its role in the control of energy balance remains unexplored. Here, using fluorescent in situ hybridization, we show that the Mes5 expresses the melanocortin-4 receptor. Consistent with MC4R activation in other areas of the brain, we found that Mes5 microinjection of the MC4R agonist melanotan-II (MTII) suppresses food intake and body weight in the mouse. Furthermore, NTS POMC-projecting neurons to the Mes5 can be chemogenetically activated to drive a suppression in food intake. Taken together, these findings highlight the Mes5 as a novel target of melanocortinergic control of food intake and body weight regulation, although elucidating the endogenous role of this circuit requires future study. While we observed the sufficiency of Mes5 MC4Rs for food intake and body weight suppression, these receptors do not appear to be necessary for food intake or body weight control. Collectively, the data presented here support the functional relevance of the NTS POMC to Mes5 projection pathway as a novel circuit that can be targeted to modulate food intake and body weight.

## 1. Introduction

The control of food intake involves complex processing by diverse nuclei of the brain [1,2,3,4]. Ultimately, feeding involves organized oromotor movements (e.g., chewing and swallowing) guided by many factors, including the constant input of sensory information from the mouth. While orosensory input is critical for consummatory ingestive behavioral responses, the regulation of food intake by orosensory neural substrates is not well understood. Work utilizing a chronic decerebrate rat model demonstrated that the isolated caudal brainstem is sufficient for the production of patterned and timing of ingestive consummatory responses, e.g., licking, chewing, and swallowing when food is placed in the oral cavity [5]. Furthermore, in response to satiation signals arising from the gastrointestinal tract and integrated within the caudal brainstem, decerebrate rats adjust their pattern of ingestive responses [6,7,8]. Together, these data suggest that the caudal brainstem circuits that integrate orosensory input to affect the pattern of oromotor output are also sensitive to homeostatic state signals. Research investigating caudal brainstem sites of action that are sufficient for adjusting oromotor patterns in response to the neural and hormonal signals that communicate nutritional status have been largely limited to structures within the dorsal vagal complex (DVC) (see, for review, [9]), although there are other caudal brainstem nodes along the sensory–motor axis of food intake control that are additional candidate sites of action for integrating satiation signals [10].

The mesencephalic trigeminal nucleus (Mes5) of the dorsal pons is a central relay site for transmitting sensory input from the oral cavity to coordinate orofacial movements of the jaw, face and neck during chewing and swallowing [11]. As the only known nucleus in the brain that contains cell bodies of primary afferent sensory neurons, the Mes5 has been described as a displaced sensory ganglion [12,13]. While it is known that the Mes5 transmits oromotor proprioceptive information—specifically, mechanical stimulation of the jaw muscles and ligaments of the teeth [14]—the ability of these neurons to also integrate metabolic information has not been well investigated. Dense innervation of peptide-like fibers that form pericellular basket-like innervation around Mes5 neurons [11,15], and that Mes5 neurons express energy balance relevant receptors (e.g., Substance P, enkephalin, histamine, galanin, cholecystokinin, calcitonin-gene related peptide, neuropeptide Y, and orexin; see [11,16] for review), suggests that the Mes5 may be sensitive to the homeostatic energy balance state.

Here, we investigate for the first time a role for Mes5 melanocortin-4 receptors (MC4Rs) in food intake and body weight regulation. MC4Rs are activated by alpha-melanocyte stimulating hormone (α-MSH), a peptide endogenously released from proopiomelanocortin (POMC) neurons uniquely expressed by neurons of the hypothalamic arcuate nucleus of (ARC) and the medullary nucleus tractus solitarius (NTS). Knockout mice for POMC or MC4R are hyperphagic and obese [17,18,19] and, in humans, mutations in the POMC and MC4R genes produce severe obesity [20,21]. Understanding POMC circuits and their contribution to energy balance regulation has been investigated for two decades [22]; however, the vast majority of these studies have examined the bidirectional modulation of ARC POMC neurons by anorectic and orexigenic hormones and their functionally relevant projections to downstream MC4R-expressing targets of known relevance to food intake and body weight regulation [23,24]. However, selective activation of either ARC or NTS POMC neurons produces anorexia [25,26]. Interestingly, while NTS POMC neurons represent one tenth of the ARC population [26,27,28], they are thought to contribute to half of the alpha-MSH immunoreactivity in the brain, with terminal fields largely restricted to the hindbrain [29]. As such, we decided to examine NTS POMC projection to the Mes5 as a potential hindbrain mechanism by which satiation signals that putatively engage the NTS POMC neurons [30,31] could ultimately affect the oral sensory–motor process of feeding. Using pharmacological and virus-mediated knockout strategies in the mouse, we found that MC4Rs are expressed on Mes5 neurons, identified using fluorescent in-situ hybridization (FISH), and are sufficient but not necessary for food intake control. We go on to show that NTS to Mes5 POMC projections can be chemogenetically activated to suppress feeding, thereby supporting the functional relevance of a previously undocumented circuit for food intake control.

## 2. Materials and Methods

### 2.1. The Animals

Adult (8 weeks old) male and female mice were used as subjects of this study. Strains of mice used included POMC-Cre^+^ for chemogenetic studies and MC4R^flox/flox^ for pharmacological and MC4R knockout studies, both obtained from Jackson Laboratories (stock no. 005965 and 023720, respectively). Animals were group housed on a reverse 12 h/12 h light/dark cycle (lights off at 10:00 a.m.) in a temperature (20–22 °C) and humidity (45–55%) controlled vivarium with ad libitum access to tap water and chow (LabDiet Rodent 5001, LabDiet, St. Louis, MO) unless noted otherwise. Following surgical preparation, mice were single-housed. All of the procedures were conducted in strict adherence to the National Institutes of Health *Guide for the Care and Use of Laboratory Animals* and were approved by the Institutional Animal Care and Use Committee of The University of Pennsylvania.

### 2.2. Fluorescence In Situ Hybridization (FISH)

Brains were removed and snap-frozen in dry, ice-cold hexane. Twenty-µm-thick coronal sections containing the Mes5 were obtained using a cryostat and stored at −80 °C until further processing. FISH was performed using a commercially available kit (Cat. No. 320,851 RNAscope Fluorescent Multiplex Detection Reagent Kit, Advanced Cell Diagnostics) according to the manufacturer’s instructions. Briefly, sections were fixed in 4 °C 10% NBF for 15 min. Slides were washed 2× with 1× PBS and dehydrated in ascending ethanol solutions (5 min washes in 50, 70, 100, 100% ethanol). After the second 100% ethanol wash, slides were air-dried, and a hydrophobic barrier was created around the sections. Slides were treated with Protease IV and incubated for 30 min at room temperature. Next, slides were rinsed with 1× PBS twice and then incubated for 2 h at 40 °C using ACD probes for MC4R, parvalbumin and dopamine beta-hydroxylase mRNA or a negative or positive control probe. Following incubation, slides were rinsed twice with 1X wash buffer before being treated with a series of amplification steps at 40 °C: 30 min incubation in AMP1-FL, 15 min incubation in AMP2-FL, 30 min incubation in AMP3-FL, 15 min incubation in AMP4-FL (with two rinses in wash buffer between each step). Following the amplification steps, the sections were counterstained with DAPI and coverslipped with ProLong Gold Antifade Mountant. Slides were stored at 4 °C and imaged 24 h later using the negative control probe-treated sections to correct for background fluorescence.

### 2.3. Stereotaxic Surgery Preparation

Mice were anesthetized by isofluorane inhalation and prepared with either a bilateral infusion guide cannula, a virus infusion or both.

For pharmacological agonism (*n* = 23; *n* = 14 males, *n* = 9 females) or antagonism (*n* = 20; *n* = 12 males, *n* = 8 females) of the Mes5 MC4R, mice were prepared with a bilateral guide cannula (1.5 mm spacing, 26-gauge; Plastics One, Roanoke, VA, USA) positioned above the Me5 according to the following stereotaxic coordinates informed by the mouse atlas of Franklin and Paxinos [32]: 5.4 mm posterior to bregma, ±0.75 mm medial/lateral, 1.2 mm dorsal/ventral from the skull surface. Jeweler screws and dental cement were used to affix the guide cannula to the skull.

For virus-mediated MC4R knockout, the Mes5 of MC4R^flox/flox^ mice was targeted with an infusion guide cannula (see above for coordinates). Mice were either injected bilaterally (100 nl/hemisphere; 10 nl/sec) with an adeno-associated virus (AAV) expressing GFP (AAV-GFP; titer = 5 × 10^12^; *n* = 7) or Cre-recombinase (AAV-Cre-GFP; titer = 5 × 10^12^; *n* = 11). Injectors were left in place for 2 min to allow for diffusion of the drug. In these mice, guide cannulas were removed following virus delivery and animals were sutured. All mice were given subcutaneous analgesia (Metacam; 5 mg/kg, Boehringer Ingelheim Vetmedica, St Joseph, MO, USA) immediately following surgery and for three additional postoperative days.

For chemogenetic experiments, POMC-Cre^+^ mice were used. Mice were prepared with a control (AAV8-hSyn-DIO-mCherry; Addgene; *n* = 21; n = 12 for IP CNO and *n* = 9 for Mes5 CNO) or an excitatory Cre-dependent designer receptor exclusively activated by designer drug (DREADD) virus (AAV8-hSyn-DIO-hM3D(Gq)mCherry; Addgene; *n* = 30; *n* = 21 for IP CNO and *n* = 9 for Mes5 CNO) injected through a bilateral guide cannula (1.0 mm spacing, 26-gauge; Plastics One, Roanoke, VA) positioned over the NTS (7.4 mm posterior to bregma, ±0.5 mm medial/lateral, 2.25 mm dorsal/ventral from the skull surface) using a micropump-depressed (PHD 2000; Harvard Apparatus, Holliston, MA, USA) Hamilton syringe attached to tubing that terminated in a 33-gauge injector extending 2.0 mm beyond the cannula. For experiments in which central clozapine-N-oxide (CNO) was delivered to the Mes5 (*n* = 18), POMC-Cre^+^ mice were also prepared with a Mes5-directed guide cannula as previously described. When CNO was delivered intraperitoneally (IP; *n* = 33), mice were only prepared with one of the two NTS-directed viruses.

### 2.4. Pharamacological or Chemogenetic Manipulations

Mice were handled and habituated to injections prior to testing. All experiments consisted of a within-subjects counterbalanced design with drug treatments separated by 72 h. At the time of or 1 h prior to DVC drug injection for Shu9119 and MTII experiments, respectively, food was removed from the animal’s cage. A micropump-depressed (PHD 2000; Harvard Apparatus, Holliston, MA, USA) Hamilton syringe attached to tubing that terminated in a 33-gauge injector extending 2.0 mm beyond the cannula was used to bilaterally deliver either artificial cerebral spinal fluid (aCSF; vehicle) or MTII (0.05 nmol; Phoenix Pharmaceuticals) to the parenchyma of the Mes5. Similarly, mice were used for injection of either aCSF or Shu9119 (60 pmol; Phoenix Pharmaceuticals) to the Mes5. The dose of Shu9119 was selected to be subthreshold for food intake and body weight effects when injected into the ventricle [5]. While the chosen dose of MTII is a low paranchymal dose [6,7], it is slightly suprathreshold for food intake suppression when directed to the fourth ventricle of the rat [8]; histological verification was used to exclude animals with postmortem ink spread to the 4th ventricle from analysis.

For central chemogenetic manipulation (*n* = 18), vehicle (0.5% DMSO) or CNO (1 mM CNO in 0.5% DMSO) was delivered similarly to the Mes5. All Mes5-delivered infusions were administered just prior to the onset of the dark cycle at a volume of 100 nl/hemisphere and a flow rate of 10 nl/sec. Injectors were left in place for 1 min to allow for diffusion of the drug.

For experiments involving peripheral administration of CNO (*n* = 33), IP injections of vehicle (0.5% DMSO) or CNO (1 mg/kg in 0.5% DMSO) were administered at the onset of the dark cycle. Food intake and body weight changes for all experiments were measured as described below. The chosen doses of CNO have been shown to be without locomotor-stimulating effects when delivered to the brain [33,34,35,36] or periphery [37].

### 2.5. Food Intake Measurements

Following pharmacological or chemogenetic manipulation at the onset of the dark cycle, mice were placed in absorbent paper-lined cages with ad libitum access to food. Food intake measurements were performed by calculating the cumulative change in the weight of the food (+/−0.001 g) from the time of injection to the indicated time point (1, 3, 6, 24 h). Food spillage was accounted for by subtracting the weight of crumbs, collected from the bottom of the absorbent paper-lined cage, from the weight change in the food pellets. Animals were weighed prior to drug treatment and at 24 h post-treatment for the calculation of body weight change. Following experimentation, placements of cannula within the Mes5 were confirmed postmortem by injection of blue dye (100 nl, 2% Chicago sky blue ink) through the guide cannula. Brains were removed and sliced on a cryostat at 30 μm. Animals with dye confined to the Mes5 were included in the analyses (Supplementary Figure S1a). For chemogenetic experiments, only mice with viral transfection of NTS POMC neurons, as evidenced by mCherry-expressing neurons, were used in analysis (Supplementary Figure S1c).

For MC4R-knockout mice, daily food intake and body weight readings began 2 days prior to and continued for 16 days following virus delivery. Following experimentation, brains were removed and sliced on a cryostat at 30 µm. Animals with GFP confined to the Mes5 (Supplementary Figure S1b) were included in the analysis.

### 2.6. Statistical Analysis

All data are expressed as means ± SEM. The sample size was chosen based on the published literature. Paired or unpaired Student’s two-tailed *t*-tests were used for statistical analysis of each time point using the GraphPad Prism 7.0 Software (GraphPad Software Inc., San Diego, CA, USA). *p* values of <0.05 were considered statistically significant.

## 3. Results

### 3.1. Parvalbumin Neurons of the Mes5 Express the MC4R

**Figure 1:**
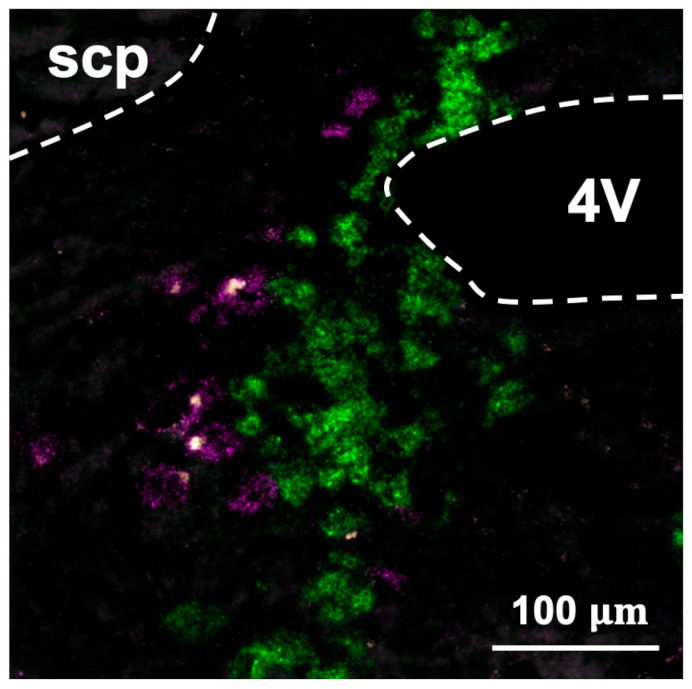
Fluorescence in-situ hybridization images demonstrating expression of the MC4R on Mes5 neurons. Colocalization indicates expression of the MC4R (yellow) on Mes5 parvalbumin-expressing neurons (magenta) that border the dopamine beta-hydroxylase-expressing neurons (green) of the locus coeruleus. 4V = fourth ventricle, scp = superior cerebellar peduncle. Image corresponds to plate level 76 of Paxinos and Watson’s Mouse Brain Atlas, fourth edition.

FISH was performed to identify expression of the MC4R on Mes5 neurons. Expression of mRNA transcripts for the MC4R (yellow) was observed on Mes5 neurons that express parvalbumin (magenta) but not dopamine-beta-hydroxylase-expressing neurons (green) of the adjacent locus coeruleus (Figure 1).

### 3.2. Pharmacological Activation of Mes5 MC3/4Rs Suppresses Food Intake and Body Weight in Male and Female Mice

**Figure 2:**
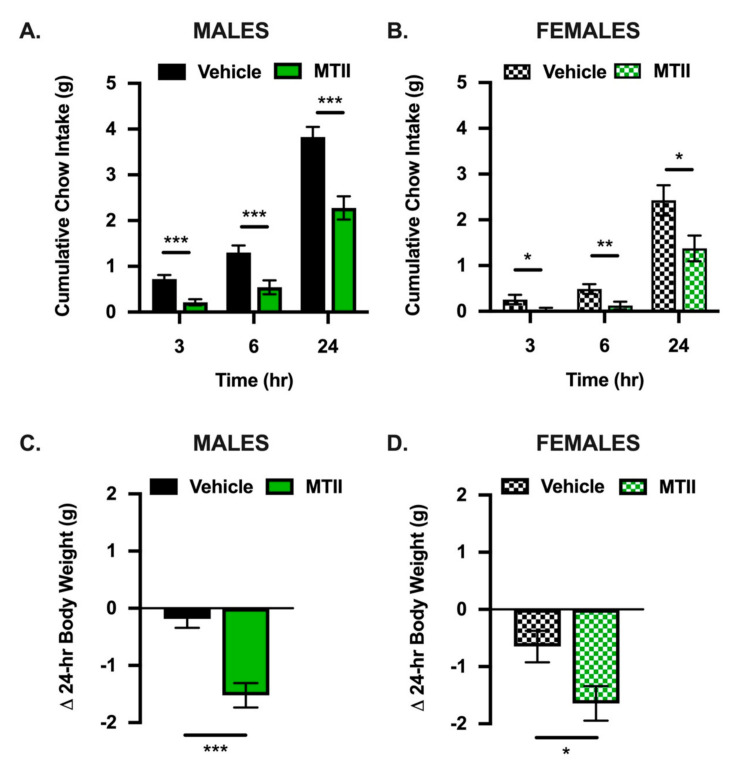
Pharmacological activation of Mes5 MC4Rs suppresses food intake and body weight in male and female mice. (**A**,**B**) Bilateral Mes5 infusion of the MC3/4R agonist MTII (0.05 nmol) suppresses cumulative chow intake at 3, 6, and 24 h post-manipulation and (**C**,**D**) 24 h body weight change relative to vehicle treatment in male and female mice. Data represent means ±SEM; Paired Student’s *t*-tests: * = *p* < 0.05, ** = *p* < 0.01, *** = *p* < 0.001; *n* = 14 males, *n* = 9 females.

To test the sufficiency of Mes5 MC3/4Rs for food intake control, we compared cumulative chow intake and body weight changes following pharmacological activation of Mes5 MC3/4Rs in male and female mice using the nonselective MC3/4R agonist melanotan II (MTII). In both males and females, we observed food intake suppression by MTII at 3, 6, and 24 h (Figure 2A,B) as well as a suppression of body weight at 24 h (Figure 2C,D).

### 3.3. Mes5 MC4Rs Are Not Necessary for Food Intake Control in Male and Female Mice

**Figure 3:**
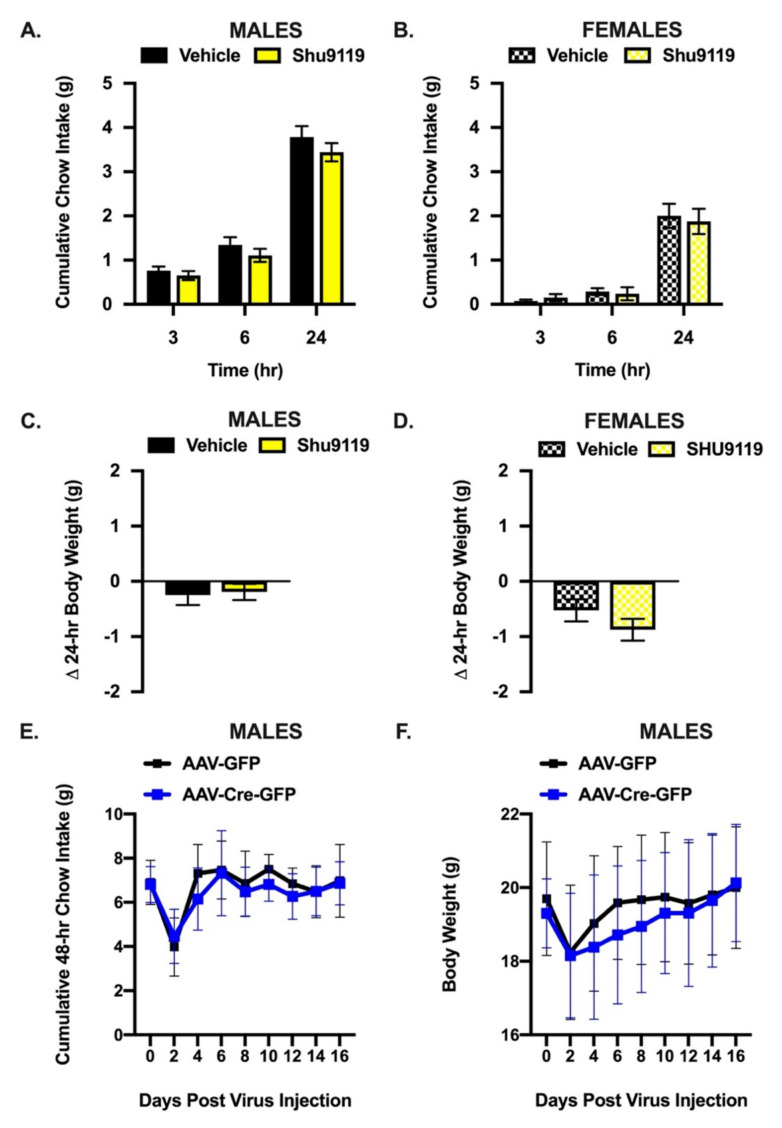
The Mes5 MC4R is not necessary for food intake or body weight control. Bilateral Mes5 infusion of the MC3/4R antagonist SHU9119 (60 pmol) does not impact cumulative chow intake in male (**A**) or female (**B**) mice. 24 h body weight is also not impacted by Mes5 directed infusion of SHU9119 in male ((**C**); *n* = 12) or female ((**D**); *n* = 8) mice. Cumulative 48-hr chow intake (**E**) and body weight (**F**) over 16 days is similar for Mes5 MC4R knockout (AAV-Cre-GFP; *n* = 11) and control (AAV-GFP; *n* = 7) mice. Data represent means ± SEM; Paired Student’s *t*-tests.

We first used a pharmacological approach to test the necessity of Mes5 MC3/4Rs for food intake control by measuring cumulative food intake and body weight changes following Mes5 delivery of either vehicle or the non-selective MC3/4R antagonist Shu9119. We did not observe group differences at any measured timepoint for either cumulative chow intake or change in body weight in male (Figure 3A,C) or female (Figure 3B,D) mice.

We next used a viral knockout approach [38] in male mice to probe the effects of chronically removing the contribution of the Mes5 MC4R to food intake control. Cumulative 48 h food intake (Figure 3E) and body weight (Figure 3F) were similar for AAV-GFP and AAV-Cre-GFP prepared MC4R^flox/flox^ mice at every measured timepoint.

### 3.4. Chemogenetic Activation of NTS POMC Neurons Suppresses Food Intake in Male and Female Mice

**Figure 4:**
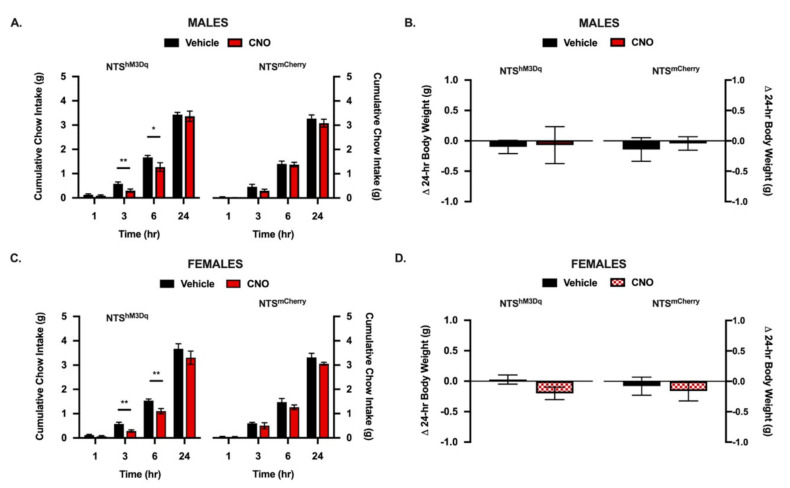
Chemogenic activation of NTS POMC projections acutely suppresses food intake. (**A**) In NTS^hM3Dq^ male, but not NTS^mCherry^ control male mice, injection of CNO (1 mg/kg; IP) suppresses 3 and 6 h cumulative chow intake but not 1 or 24 h food intake or (**B**) 24 h body weight change relative to vehicle treatment. (**C**) In NTS^hM3Dq^ female, but not NTS^mCherry^ control female mice, CNO suppresses 3 and 6 h chow intake but not 1 or 24 h food intake or (**D**) 24 h body weight change relative to vehicle treatment. All data represent means ± SEM; Paired Student’s *t*-tests: * = *p* < 0.05, ** = *p* < 0.01; *n* = 10 male NTS^hM3Dq^ and *n* = 7 male NTS^mCherry^, *n* = 11 female NTS^hM3Dq^ and *n* = 5 female NTS^mCherry^.

Activation of NTS POMC neurons using a DREADD approach has been shown to acutely suppress food intake [26]. To validate our mouse model, replicate these findings in male mice, and explore the contribution of NTS POMC neurons to feeding in female mice, we used a Cre-dependent adeno-associated virus (AAV) to express the gene encoding the evolved human M3-muscarinic receptor (hM3Dq) selectively in NTS POMC neurons (NTS^hM3Dq^). We measured food intake and body weight changes following intraperitoneal (IP) delivery of vehicle or the hM3Dq ligand CNO in male and female mice. Consistent with previous reports, in NTS^hm3Dq^ mice, IP CNO dramatically reduced cumulative chow intake at early time points (3 and 6 h; Figure 4A) relative to vehicle injection. There were no differences in 24 h chow intake (Figure 4A) or body weight at 24 h (Figure 4B) when comparing CNO to vehicle-treated mice. Importantly, no differences in food intake or body weight were observed in mice injected with a control mCherry-expressing virus (NTS^mCherry^; Figure 4A,B), indicating that the deceases in food intake are not attributed to nonspecific effects of CNO. In female mice, we observed food intake suppression by IP CNO in NTS^hm3Dq^, but not NTS^mCherry^ mice, at 3 and 6 h (Figure 4C). There were no differences in 24 h chow intake (Figure 4C) or body weight at 24 h (Figure 4D) when comparing CNO to vehicle-treated mice. No differences were observed in food intake (Figure 4C) or body weight (Figure 4D) in NTS^mCherry^ mice.

### 3.5. Chemogenetic Activation of NTS POMC to Mes5 Projections Suppresses Food Intake in Male Mice

**Figure 5:**
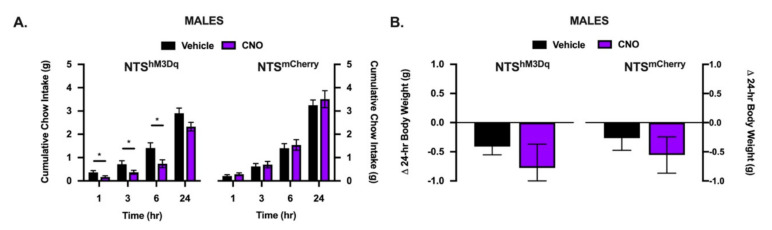
Chemogenic activation of NTS POMC projections to the Mes5 acutely suppresses food intake. (**A**) In NTS^hM3Dq^ mice, but not NTS^mCherry^ control mice, injection of CNO (1 mM) into the bilateral Mes5 suppresses 1, 3, and 6 h cumulative chow intake but not 24 h food intake in NTS or (**B**) 24 h body weight change relative to vehicle treatment. All data represent means ± SEM; Paired Student’s *t*-tests: * = *p* < 0.05; *n* = 9 NTS^hM3Dq^ and *n* = 9 NTS^mCherry^.

To determine whether NTS POMC projections to the Mes5 are sufficient for food intake suppression, we analyzed food intake and body weight following administration of CNO to the Mes5 of POMC-Cre^+^ mice prepared with an excitatory Cre-recombinase-dependent virus delivered to the NTS. Only male mice were used as no sex differences were observed when all NTS POMC projections were activated by IP CNO (Figure 4). Local infusions of CNO to the projection targets of excitatory or inhibitory DREADD-expressing neurons have been shown to manipulate behavioral responses [33,34,35,36,39]. Here, we use this approach to selectively excite only Mes5-projecting NTS POMC neurons. In NTS^hm3Dq^ mice, cumulative chow intake was suppressed for CNO relative to vehicle injected POMC-Cre^+^ mice at 1, 3, 6 h (Figure 5A). Neither chow intake (Figure 5A) nor body weight (Figure 5B) were changed at 24 h post Mes5 CNO. Importantly, no differences in food intake (Figure 5A) or body weight (Figure 5B) were observed in NTS^mCherry^ mice.

## 4. Discussion

Neural and hormonal communicators of the physiological state regulate aspects of energy balance control including the sensory–motor coordination of oral motor consummatory behavior required for food intake. By acting on multiple nuclei that modulate sensory input and motor output during feeding, metabolic signals have been shown to modify orosensory information (i.e., taste) and influence the coordination of licking, chewing, and swallowing [40,41]. Here, we identify a role for the Mes5, a node in orosensory–motor control, in food intake regulation. We show that the Mes5 expresses MC4Rs that can be activated to suppress food intake and body weight in male and female mice. Furthermore, we demonstrate that NTS POMC projections to the Mes5 can be chemogenetically activated to suppress food intake and are therefore likely to be among the hindbrain circuits that are sufficient for altering ingestive oromotor responses in response to satiation signals [42,43].

To the best of our knowledge, there have been very few other documented instances of food intake regulation via the Mes5 [44,45,46]. We expect that the Mes5 has been overlooked because of its anatomically ambiguous nature. The Mes5 consists of a very small cluster of neurons that are distributed across a long rostral caudal axis of the dorsal portion of the pons. The Mes5 has no defined borders and, without staining for a cellular marker of Mes5 neurons like parvalbumin, as done here, Mes5 cells are continuous with those of the locus coeruleus, periaqueductal grey, parabrachial nucleus, and laterodorsal tegmental nucleus at particular rostral/caudal levels of the brain. Often, the area where Mes5 neurons are located is referred to as the pre- or peri-locus coeruleus, with no effort to distinguish Mes5 neurons cytochemically [47,48]. It is worth noting that many of the aforementioned nuclei have well-documented roles as CNS nuclei of relevance to food intake control [49,50,51,52,53]. Thus, it is possible that prior work targeting these structures in assessing feeding behavior may have also inadvertently affected the neural processing of the Mes5.

Here, we use FISH to show the MC4R on Mes5 neurons, defined by their expression of parvalbumin. While we only observed a subset of Mes5 neurons to be MC4R+, pharmacological activation of MC4R-expressing Mes5 neurons was sufficient for the suppression of food intake (Figure 2A) and body weight (Figure 2B). Expression patterns of the melanocortin-3-receptor should be examined in future studies, as MTII is also an agonist for the MC3R. The feeding effects produced by agonism of a limited population of MC4R+ neurons are robust, have a rapid onset, and raise consideration of the phenomenon of electrical coupling of neurons via gap junctions, which were first reported in Mes5 neurons [54,55]. The impact of MC4R stimulation may be amplified by the generation of action potentials in adjacent coupled cells, although the impact of synchronization of Mes5 neurons on orofacial movement, and feeding behavior more specifically, is unknown [56,57].

The neural circuits controlling the rhythmic coordination of chewing are not as well understood as other motor aspects of feeding like swallowing [58]. However, the Mes5 is believed to function as a premotor nucleus for the control of mastication [59] via projections to diverse structures that collectively control oromotor output during feeding. Axons of Mes5 neurons pass peripherally through all three subdivisions of the trigeminal nerve, including those that innervate the spindles of jaw closer muscles and mechanoreceptors of periodontal ligaments [60,61,62]. Centrally, the Mes5 axons send branches to motoneurons and preganglionic neurons (e.g., the supratrigeminal region, trigeminal motor nucleus, and the principle trigeminal nucleus and hypoglossal nucleus) [63,64] and innervate caudal brainstem nuclei with documented roles in feeding behavior. These nuclei include the NTS, inferior olive, cranial nerve motor nucleus, locus coeruleus, pontine supratrigeminal zone, deep tectal grey, and midbrain reticular formation [65]. Mes5 modulation of these structures and feeding behavior has been largely unexplored [66]; however, ascending Mes5 projections to the histaminergic neurons of the tuberomammillary nucleus [44] have been shown to play a role in energy balance control. The downstream targets of MC4R-expressing Mes5 neurons, specifically, remain to be determined but would greatly inform a potential circuit underlying the food intake suppression following Mes5 MC4R activation.

Our data suggest that the endogenous source of ligand for the MC4R is coming from NTS POMC neurons. While previous tracing work failed to examine projections to the Mes5 specifically, there have been observations of POMC terminals in the Mes5 of the rat [67]. Furthermore, selective activation of an NTS POMC to Mes5 projection was sufficient to suppress food intake (Figure 5). Unlike pharmacological activation of Mes5 MC4Rs, activation of all NTS POMC projections or specifically those to the Mes5 did not suppress body weight. We hypothesize that this is due to the short half-life of CNO relative to MTII. It is also possible that chemogenetic activation of NTS POMC neurons is activating competing circuits that attenuate food intake suppression, perhaps via the release of the inhibitory neurotransmitter GABA, which is known to be co-released from POMC neurons along with alpha-MSH [68]. Future work will be necessary to disentangle the nature of the discrepancy in the time-courses of our observed effects. Furthermore, while arcuate POMC projections to the dorsal pons were not revealed in whole brain mapping of POMC projections in the mouse [29], the possibility of arcuate POMC modulation of feeding behavior via Mes5 MC4R activation cannot be excluded. Parallel experiments are required to determine if the effects that we observed are exclusive to the hindbrain-restricted circuit that we chose to examine here.

While the identification and functional relevance of this particular NTS POMC-to-Mes5 projection pathway is novel, projections from the NTS to an extensive list of downstream structures have a well-documented role in food intake control. Indeed, the NTS serves as a hub for the integration of neural and humoral energy status [9]. In in vitro preparations, both stimulation of the vagus nerve and application of the short-acting satiation signal cholecystokinin (CCK) activate NTS POMC neurons [30,31]. Whether or not CCK-derived signals reach the Mes5 via an NTS POMC projection remains to be tested but is one potential mechanism by which the Mes5 MC3/4RS are engaged by satiation signals. As it has been shown that food intake suppression by CCK requires the MC4R—specifically, those within the hindbrain [31]—the necessity of Mes5 MC4Rs for CCK-induced food intake suppression should also be investigated.

We hypothesize that satiation signals are engaging NTS POMC neurons, which causes downstream activation of Mes5 MC4Rs to interfere with Mes5 signaling and suppress aspects of normal oromotor control, thereby causing the suppression of food intake that we have observed. Our hypothesis is consistent with data showing that the medulla imparts inhibition in pontine structures that are sufficient for the expression of masticatory movements [69,70,71]. Furthermore, MTII and alpha-MSH have been shown to hyperpolarize neurons [72]. Future work will be required to characterize the neural activity changes in Mes5 and downstream structures following NTS POMC neurons and Mes5 MC4R activation. Additionally, studies using detailed measures of oromotor output, such as EMG recordings of the masseter [73,74,75], lick microstructure [76], or taste reactivity analysis [77], will be necessary to reveal the mechanism by which MC4R activity on Mes5 neurons interferes with normal feeding. It would also be interesting to investigate the impact of Mes5 MC4R deletion on oromotor output. In our studies, Mes5 MC4Rs were not necessary for 48 h food intake or body weight control. There are redundant circuits of the brain that contribute to cumulative food intake control and perhaps the role of the MC4R on Mes5 neurons is nuanced such that cumulative food intake control is not impacted while aspects of feeding are altered. This idea is supported by data in which Mes5-lesioned mice have unaffected cumulative food intake and body weight [44] but have disrupted feeding patterns such that lesioned mice spend more time in the food chamber when food is available but make fewer entries into the food chamber compared to sham-operated mice [44]. It is possible that the disruptions in oromotor output due to Mes5 involvement go unseen until a detailed analysis of oromotor output is conducted. Lastly, our studies were limited to food intake and, as such, the potential impact of Mes5 MC4R stimulation on energy expenditure, respiratory quotient, or oxidative metabolism remains unknown.

The sufficiency of NTS POMC neurons to suppress food intake (see [26] and Figure 4) warrants investigations of other NTS POMC projections and their relevance in food intake control. Here, we identify the Mes5 as one of these novel targets, although it likely represents one of many NTS POMC projections that subserve the oral motor control of ingestive behavior. NTS POMC neurons densely innervate other discrete hindbrain nuclei known to regulate food intake and oral motor outputs, including the multiple subdivisions of the reticular nucleus, lateral parabrachial nucleus, supratrigeminal nucleus, and motor trigeminal nucleus [29]. NTS POMC projections to these structures with a demonstrated role in the coordination of chewing and swallowing by these NTS POMC projections warrant investigation. Likewise, future studies should interrogate the role of other feeding peptide receptors within the Mes5 (see [11,16] for review) for their control of feeding via satiation signal-derived modulation of orosensory–oromotor communication. Elucidating the sources of these peptides and whether targeting these receptors in the Mes5 can alter food intake remains to be investigated. A comprehensive knowledge of the involvement of feeding peptides in the Mes5 may be beneficial for advancing our understanding of the neural control of normal feeding behavior, as well as the pathology of sensory-based feeding disorders [78] and other psychiatric diseases with known oral sensory–motor deficits, such as autism [79,80], Rett syndrome [81], and Prader–Willi syndrome [82].

## Data Availability

The data presented in this study are available on request from the corresponding author.

## Supplementary Materials

The following are available online at https://www.mdpi.com/article/10.3390/nu13051642/s1, Figure S1: Histological verification of pharmacological and viral targeting of the Mes5 and NTS. (a) Representative image of ink placement in the Mes5 representing the site of targeted by Mes5-directed drugs including vehicle, MTII, Shu9119 and CNO. (b) Representative image of AAV-Cre-GFP and AAV-GFP injection into the Mes5 of MC4R^flox/flox^ mice. (c) Representative image of NTS-directed AAV8-hSyn-DIO-hM3D(Gq)mCherry in POMC-Cre+ mice. 4V = fourth ventricle, scp= superior cerebellar peduncle, NTS = nucleus tractus solitarius.
