# Supplementary material for: The Mesencephalic Trigeminal Nucleus Controls Food Intake and Body Weight via Hindbrain POMC Projections
Source: Nutrients. 2021 May 13;13(5):1642. doi: 10.3390/nu13051642 (PMC8152732; doi:10.3390/nu13051642)
Supplement: Supplementary file 1 [file nutrients-13-01642-s001.zip › nutrients-1190442-supplementary.pdf]

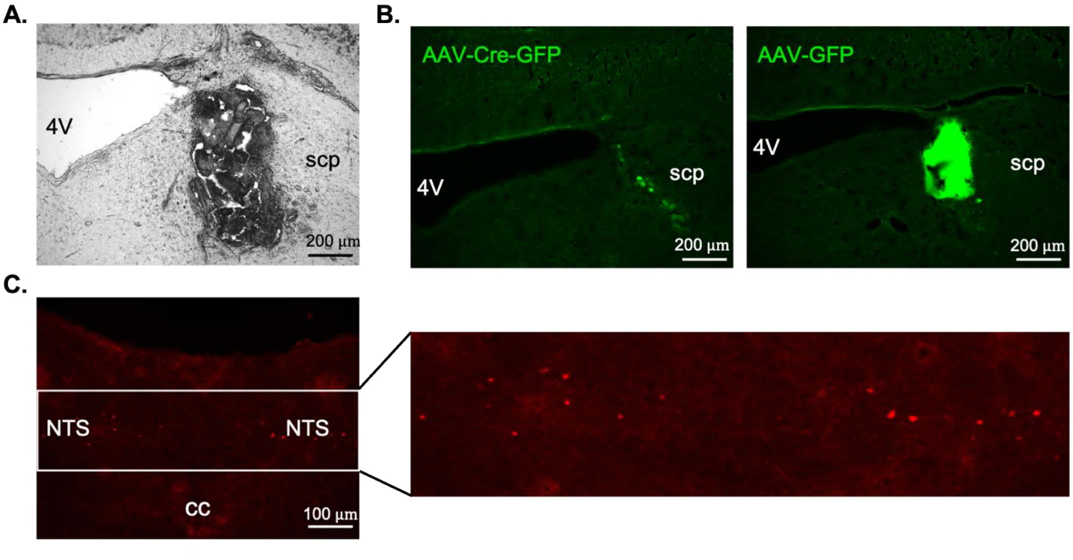

Figure S1. Histological verification of pharmacological and viral targeting of the Mes5 and NTS. (a) Representative image of ink placement in the Mes5 representing the site of targeted by Mes5-directed drugs including vehicle, MTII, Shu9119 and CNO. (b) Representative image of AAV-Cre-GFP and AAV-GFP injection into the Mes5 of MC4R$^{flox/flox}$ mice. (c) Representative image of NTS-directed AAV8-hSyn-DIO-hM3D(Gq)mCherry in POMC-Cre^{+} mice. 4V= fourth ventricle, scp= superior cerebellar peduncle, NTS=nucleus tractus solitarius.
